# Chronic dizziness: neuro-sensitization and psycho-sensitization

**DOI:** 10.3389/fnint.2026.1929481

**Published:** 2026-09-24

**Authors:** Moyan Wang, Qi Han, Xin Teng, Dingyu Liu, Zhen Yang, Huiying Li, Yingxia Yu, Wei Zhao, Ke Xiang, Li Sun

**Affiliations:** 1 College of Traditional Chinese Medicine, Changchun University of Chinese Medicine, Changchun, China; 2 Department of Geriatrics, Dizziness Clinic, Jilin Academy of Chinese Medicine Sciences, Changchun, China; 3 Institute of Pharmacology and Toxicology of Traditional Chinese Medicine, Jilin Academy of Chinese Medicine Sciences, Changchun, China

**Keywords:** chronic dizziness, neuro-sensitization, PPPD, psycho-sensitization, vestibular vertigo, VM

## Abstract

This review aims to investigate the potential mechanisms driving the transition from acute vestibular events to chronic dizziness, focusing on the roles of neural sensitization and psychological sensitization in symptom persistence. The evolution of vestibular dizziness is analyzed from the perspectives of vestibular compensation, sensory reweighting, and the fear-avoidance model. After acute vestibular injury, central nervous system function can be partially restored through vestibular compensation, sensory reweighting, and synaptic plasticity. However, this recovery process is often accompanied by increased visual dependence, which provides a foundation for the development of neural sensitization. The abnormal integration of proprioceptive and visual input described by the fear-avoidance model not only contributes to central sensitization but also reveals the core feature of psychological sensitization: perceptual amplification driven by catastrophizing cognition and avoidance behavior. Patients with chronic dizziness exhibit hypersensitivity to visual and proprioceptive stimuli and reduced sensory processing thresholds, which reflects abnormal sensory gain at the level of neural sensitization. Psychological factors, in turn, maintain or exacerbate neural sensitization by modulating proprioceptive processing and limbic system activity. Neural sensitization and psychological sensitization mutually reinforce each other, constituting a critical link in the transition from acute vestibular events to chronic dizziness. Comprehensive interventions targeting sensory reweighting, fear-avoidance behavior, and psychiatric comorbidity may help interrupt this process.

## 1 Introduction

Vertigo is a common clinical symptom, with vestibular vertigo accounting for approximately one quarter of vertigo complaints. One category consists of those with clear organic lesions, such as otolith displacement in benign paroxysmal positional vertigo (BPPV) and neuroinflammation in vestibular neuritis; the other category, with more complex etiologies, can be tentatively summarized as functional, such as ion channel abnormalities and increased cortical excitability in vestibular migraine (VM) (Neuhauser, 2016). It is noteworthy that, after experiencing similar acute vestibular injury, some patients’ symptoms self-limit following vestibular compensation, while others develop persistent postural-perceptual dizziness (PPPD) or other forms of chronic functional impairment. Vestibular disorders are associated with physical manifestations, including vertigo and postural instability, and they substantially diminish patients’ quality of life, daily functioning, and social engagement (Molnár et al., 2022). Additionally, these disorders often coexist with psychiatric symptoms, notably anxiety and depression, which can intensify the perceived disability and complicate clinical management. This difference in outcome suggests that the pathological mechanism of chronic dizziness is not merely vestibular dysfunction; individual differences may influence the process of chronicization. This paper attempts to differentiate neural sensitization and psychological sensitization, two concepts that are both independent and interactive, and to elucidate the role of secondary remodeling of central sensory processing and emotional circuits in the transition from acute vertigo to chronicity.

## 2 Vestibular compensation

The vestibular system participates in motor balance through the vestibulo-ocular reflex (VOR), vestibulospinal reflex (VSR), and the inputs and outputs of multiple receptors (Wallace and Lifshitz, 2016), achieving the perception of head movement and gravity changes and postural control (Kingma and van de Berg, 2016; Table 1). Hair cells in the peripheral vestibular organs convert mechanical stimuli into neural signals, and the bipolar neurons in the vestibular ganglion (Scarpa’s ganglion) transmit peripheral signals to the brainstem vestibular nuclear complex. After this, Neural signal is projected from the vestibular nuclear complex to the cerebellum, thalamus, and Cortex, participating in posture regulation, eye movement control, and Spatial cognition.

**TABLE 1:** Vestibular and cervical reflexes: sensory inputs, motor outputs, and postural consequences.

Reflex	Abbr.	Sensory input source	Motor output target	Primary function	Postural/gait impact if impaired	Clinical assessment method
Vestibulo-ocular reflex	VOR	Semicircular canals (angular acceleration)	Extraocular muscles	Stabilizes gaze during head motion	Blurred vision, oscillopsia, difficulty reading	Video Head Impulse Test (vHIT), caloric test, rotational chair
Vestibulo-spinal reflex	VSR	Utricle and saccule (linear acceleration/gravity)	Trunk and lower limb extensors/flexors	Maintains upright posture, counteracts gravity	Postural instability, increased fall risk, excessive sway while standing	Posturography, Romberg test
Vestibulo-collic reflex	VCR	Semicircular canals, otolith organs	Neck muscles (sternocleidomastoid, trapezius, etc.)	Stabilizes head relative to trunk during motion	Poor head control, exacerbation of dizziness during head turns	Head impulse test (cervical)
Cervico-ocular reflex	COR	Neck proprioceptors	Extraocular muscles	Assists gaze stabilization during trunk movement (compensates for VOR)	Insufficient compensation when VOR is impaired, reduced dynamic visual acuity	Dynamic visual acuity test, head thrust
Cervico-spinal reflex	CSR	Neck proprioceptors	Trunk and upper limb muscles	Adjusts trunk muscle tone according to cervical posture	Abnormal trunk compensation, altered gait	Postural sway analysis
Cervico-collic reflex (cervico-cervical reflex)	CCR	Neck proprioceptors	Contralateral neck muscles	Coordinates bilateral neck muscle tone, maintains head centering	Neck muscle tone imbalance, head tilt	Clinical observation, EMG

After unilateral vestibular injury, the activity of ipsilateral vestibular nucleus neurons significantly decreases—a state of deafferentation—leading to asymmetry of static and dynamic vestibular reflexes, manifesting as spontaneous nystagmus and postural deviation. The medial vestibular nucleus (MVN) contains two main types of neurons: A-type neurons and B-type neurons. A-type neurons have a wide action potential and a monophasic afterhyperpolarization, whereas B-type neurons have a narrow action potential and a biphasic afterhyperpolarization (Faulstich et al., 2006). Johnston et al. (1994) found that, after unilateral labyrinthectomy (UL), the intrinsic membrane properties of B-type MVN cells undergo significant changes, and their spontaneous firing rate begins to recover as early as 4 h after denervation, a process known as compensatory increase in excitability (CIE). This deafferentation model highlights the clinical significance of preserving inner ear structures whenever feasible, as destructive interventions may undermine central compensatory mechanisms and increase the risk of chronic dizziness in patients. This difference may be related to the differential expression of ion channels on different types of neurons. Van Welie and du Lac (van Welie and du Lac, 2011) further revealed that large-conductance calcium-activated potassium channels (BK channels) are bidirectionally regulated by Ca2+/calmodulin-dependent protein kinase II (CAMKII) and protein kinase C (PKC); CAMKII increases the open probability of BK channels by shifting the voltage activation range toward hyperpolarization, whereas PKC decreases it, thereby dynamically regulating the excitability of MVN neurons. During the first 3 days after unilateral vestibular input loss, the expression of potassium-chloride cotransporter 2 (KCC2) in the ipsilesional vestibular nucleus (VN) is significantly downregulated, leading to excessive intracellular Cl- concentration in MVN neurons; at this time, presynaptic GABA is released and acts on GABAA receptors on MVN neurons, and channel opening causes intracellular Cl- efflux (Dutheil et al., 2016). The reversal or attenuation of this GABAergic inhibition helps restore neuronal excitability on the affected side. In the UL rat model, histamine H1 receptor-mediated asymmetric activation of the commissural inhibitory system is manifested as a selective upregulation of postsynaptic H1 receptors on the affected side, which increases neuronal excitability on the affected side and, via the commissural inhibitory pathway, inhibits excitability on the intact side, thereby balancing bilateral vestibular nuclear (VN) electrical activity (Chen et al., 2019). Protein kinase C-mediated phosphorylation reduces conductance, whereas activation of calmodulin kinase II increases it.

Asymmetric excitation and recovery processes drive vestibular compensation, encompassing both static and dynamic compensation. Static compensation denotes the rebalancing of resting discharge activity in the bilateral vestibular nuclei when the head is stationary, a process typically completed shortly after injury. Dynamic compensation refers to the functional recovery of the VOR and vestibulospinal reflex during head movements; this recovery is slow and often incomplete (Takeda et al., 2024). During static compensation, the ocular tilt reaction—i.e., ocular motor signs (spontaneous vestibular nystagmus, skew deviation, ocular torsion), postural signs (head and body tilt toward the lesion side), and perceptual signs (vertigo, subjective visual vertical tilt)−appears simultaneously. Acute unilateral vestibular deafferentation leads to a severe imbalance in resting discharge within the bilateral vestibular nuclear complex. In the same MVN, a decrease in the spontaneous discharge rate and sensitivity of type I VN neurons is observed, while the enhanced inhibitory drive from the contralateral side via inhibitory commissural pathways further exacerbates this imbalance. Dynamic recovery is slower, and the complete compensation of static eye movements and postural disorder is due to the rapid rebalancing of spontaneous Resting Discharge in the bilateral VN complexes (Lacour et al., 2016). During late static compensation after UL, the resting activity of ipsilateral vestibular nucleus neurons gradually recovers without the need to inhibit contralateral vestibular nucleus neurons. During dynamic vestibular compensation, changes such as long-term potentiation of synapses and commissural sprouting lead to the recovery of sensitivity of ipsi-MVe neurons to head velocity and acceleration, thereby restoring the dynamic vestibulo-ocular reflex and vestibulospinal reflex (Takeda et al., 2024). Specifically, deafferentation-induced plasticity after UL involves early rebalancing mechanisms, primarily occurring in the vestibular nucleus. In the acute phase, glucose metabolic imbalance is observed in the vestibulocerebellum (including the vestibular nucleus), thalamus, multisensory vestibular cortex, hippocampus, and amygdala. During early compensation, rebalancing occurs between the vestibular nucleus, thalamus, and temporoparietal cortex, and bilaterally increased glucose metabolism is observed in the hippocampus and amygdala (Zwergal et al., 2016).

Imaging findings from voxel-based morphometry (VBM) and resting-state functional MRI (rs-fMRI) studies after unilateral vestibular disorder revealed changes in gray matter volume (GMV) in the somatosensory cortex, multisensory vestibular cortex areas, namely the superior temporal gyrus (STG), insula, inferior parietal lobule (IPL), middle temporal area (MT/V5), posterior hippocampus, and at the level of the gracile nucleus and vestibular nuclei in the brainstem (Lacour et al., 2016). Studies show that Brevican, a component of the perineuronal net in the superior vestibular nucleus, undergoes changes in expression after injury, indicating that extracellular matrix remodeling around neurons participates in vestibular compensation (Magyar et al., 2022).

Other studies conducted in animal models have demonstrated that histological damage to the vestibular end organs is not always permanent. In cases of ischemia or moderate ototoxicity, which primarily affect the primary synapses of the inner ear, spontaneous synaptic repair occurs (Tighilet et al., 2019). During this period, some surviving synaptic contacts within the vestibular sensory organs and sensory input from the contralateral vestibular system participate in the compensation process. Of course, despite some evidence from animal experiments, complete destruction of the unilateral vestibular system is uncommon in the actual disease course. Although UL provides an extreme model of central rebalancing after denervation, human chronic vestibular disorders are often not complete denervation, but rather a mixture of incomplete injury and persistent multisensory integration dysfunction. Therefore, the model mainly suggests relevant pathological processes.

## 3 Sensory reweighting and visual dependence after vestibular compensation

Sensory reweighting refers to the Central nervous system’s dynamic adjustment of the relative contributions of vestibular, visual, and proprioceptive inputs to postural control and spatial orientation based on environmental conditions and the reliability of sensory inputs. Under normal physiological conditions, when one type of sensory information becomes unreliable, such as reduced Visual input in darkness or distorted Proprioception on an unstable surface the Central nervous system enhances its reliance on other sensory channels to maintain stable postural control (Peterka, 2002). The vestibular nuclear complex is a key hub for multisensory integration. Afferent signals from the peripheral vestibular organs are integrated with cervical proprioception and visual motion signals at the level of the brainstem vestibular nuclei, cerebellum, and cortex. Multisensory input converges on the vestibular nuclear complex, enabling neurons to respond to multiple types of sensory information and, when one sensory channel is impaired, to maintain function through compensation from other channels (Guilding and Dutia, 2005).

Postural control is closely linked to vestibular sensation, proprioception, and vision. Following the loss of a single signal, the vestibular complex adjusts the weights of visual, proprioceptive, and vestibular inputs through sensory reweighting mechanisms to maintain balance. Older adults rely more on proprioception than on visual and vestibular cues for postural control, which may be attributed to the age-related decline in the function of the visual and vestibular systems (Wiesmeier et al., 2015). This dependency is also evident in patients with Parkinson’s disease, who demonstrate greater midline instability during postural control, even though their reliance on ankle proprioception is similar to that of elderly fallers (Bekkers et al., 2014). Furthermore, the interaction between visual dependence and the VOR also plays an important role in postural control. Research has found that the availability of visual feedback affects the dynamic adaptability of postural control, especially in training that combines visual feedback with vestibular stimulation, which can significantly alter sensory organization and postural behavior (Appiah-Kubi and Wright, 2019). The dynamics of this sensory reweighting vary across sensory modalities; visual and proprioceptive reweighting occurs more slowly, likely because of the critical role of visual feedback in postural control (Assländer and Peterka, 2014).

Patients with bilateral vestibular dysfunction (BVF) exhibit a marked increase in postural sway during visual or proprioceptive deprivation, indicating their reliance on visual and proprioceptive inputs for compensation. When vision and proprioception are normal, postural control ability does not differ significantly from that of healthy individuals (Sprenger et al., 2017). Research shows that after a vestibular lesion, the weight of visual input increases, while the weight of vestibular input decreases, leading to increased visual dependence (Lubetzky et al., 2019). Conversely, the study found that after UL in mice, the number of activated neurons in the Somatosensory cortex increased, while the number of activated neurons in the Visual cortex showed no significant change (Kai et al., 2022). More interestingly, patients with chronic unilateral vestibular disorder exhibit reduced visual cortex activity, while simultaneously showing increased activity in brain regions associated with the sensorimotor network, suggesting that they likely compensate for inadequate visual substitution through behavioral substitution (Si et al., 2022). Patients with Motoric Cognitive Risk syndrome (MCR) exhibit hyperactive BOLD signals in the visual cortex, parahippocampal area, and dorsolateral prefrontal cortex, while motor-related areas (cerebellar lobules, supplementary motor area) show reduced BOLD activity (Nagamalla et al., 2024). This pattern suggests reorganization of the sensorimotor network; hyperactivity in the visual cortex may reflect an abnormal reliance on visual information. One month after neurotomy, the patient’s eyes-open postural control and gait/walking returned to normal. However, fine balance analysis revealed that during long-term follow-up, visual perception of vertical and ocular torsion disorders persisted. Only when visual input is cut off (eyes closed) do clinical postural disorders persist (Devèze et al., 2015). At 4 h after UL, synaptic blockade had no effect on the elevated *in vitro* discharge of MVN neurons; However, from 48 h to 1 week later, synaptic blockade could reduce the elevated activity to normal. This time course indicates that the early increase in excitability originates from deafferentation, whereas the later phase depends on a sustained increase in intrinsic excitability. Multisensory input converging onto the VN complex can explain this permanent change−when input from one sensory channel weakens, input from other channels can drive the neuron to maintain activity (Guilding and Dutia, 2005). This visual dependence renders patients more susceptible to the influence of visual motion, leading to visual vertigo (VV) in more complex environments, such as when walking in supermarket aisles or crowds, or when viewing moving scenes (Pollak et al., 2015).

## 4 Neural sensitization

Neural sensitization may be a key component of the pathological changes in the central nervous system that underlie chronic dizziness. It can be understood as an abnormal increase in the processing gain of the vestibular system and its associated networks in response to sensory input, causing normal stimuli to be perceived as abnormal vertigo. This understanding draws on central sensitization (CS) and peripheral sensitization. CS has been used to explain the symptom amplification mechanisms in various functional somatic syndromes. In clinical practice, it is often used as an explanatory label, but one must note the risk of circular explanation. However, the relationship between functional somatic disorders and complications such as cognitive decline and affective disorders is often attributed to CS (van den Broeke et al., 2018). Peripheral sensitization refers to the increased sensitivity of nociceptive neurons to afferent signals. Following peripheral nerve injury, damaged cells and inflammatory cells (such as mast cells and lymphocytes) release chemical mediators, including norepinephrine, bradykinin, histamine, prostaglandins, potassium ions, cytokines, serotonin, and neuropeptides. These cellular mediators, in turn, can sensitize nociceptors and amplify their afferent neural signals (Zheng et al., 2022). Both sensitization mechanisms involve the nervous system amplifying sensitivity as a defensive response after prolonged exposure to noxious inputs.

### 4.1 Synaptic plasticity

Synaptic remodeling might be a key reason for neural sensitization. Central nervous system plasticity plays a key role in the onset and recovery of vertigo (Mike and Tamás, 2018). For example, functional imaging studies reveal that after a vestibular lesion, the functional connectivity between the cerebral cortex and cerebellum changes, contributing to the vestibular compensation process (Conrad et al., 2020). Patients with vestibular neuritis typically manifest unilateral vestibular function loss during the acute phase; however, through central plasticity mechanisms, symptoms can gradually resolve (Bronstein and Dieterich, 2019). Perineuronal nets (PNNs) are specialized extracellular matrix (ECM) structures that enwrap neuronal cell bodies and proximal dendrites, playing a role in regulating synaptic plasticity and axonal regeneration. Degradation of PNNs may release constrained synaptic plasticity, promoting compensation while potentially leading to the formation of aberrant synaptic connections, thus providing a structural basis for sensitization (Rastoldo and Tighilet, 2024). Within the early time window following unilateral vestibular neurectomy (UVN), the Brainstem undergoes significant brain-derived neurotrophic factor (BDNF)-dependent remodeling of excitatory markers (Dutheil et al., 2016). BDNF regulates neuronal plasticity by targeting the tyrosine kinase B (TrkB) receptor; activating the BDNF/TrkB signaling pathway can increase the number of newly generated cells and astrocytes in the MVN, inferior vestibular nerve (IVN), superior vestibular nerve (SVN), and lateral vestibular nucleus (LVN), and improve the balance of GABAergic neurons and GABA A receptor (GABA_*A*_R) expression on the injured and intact sides of the MVN (Mao et al., 2021). Brain-Derived Neurotrophic Factor (BDNF) can increase the sensitivity of the nervous system by activating microglia (Zheng et al., 2022). Furthermore, after a vestibular lesion, the upregulation of brain-derived neurotrophic factor (BDNF) levels promotes the recovery of vestibular deficits and drives the balance of GABAergic neurons and GABA _*A*_ receptor expression between the lesioned and intact sides of the MVN (Mao et al., 2021). GABA is the main inhibitory neurotransmitter in the central nervous system, involved in regulating the excitability of vestibular nucleus neurons. Diazepam, as a GABA_A receptor agonist, can accelerate the initial compensation process after unilateral vestibular injury by enhancing GABAergic inhibition (Takeda et al., 2024). Furthermore, glutamate acts as an excitatory neurotransmitter and is involved in synaptic transmission and plasticity of vestibular nucleus neurons. For example, following UL, glutamate receptor expression and function undergo changes that contribute to vestibular compensation (Wu et al., 2024). Inhibition neurite outgrowth and excitatory synapse formation is considered an important mechanism of CS (Zheng et al., 2022). In order to suppress the bilateral vestibular sensation asynchrony caused by excitatory discharge, endogenous protein kinase C chronically inhibits the excitability of MVN neurons through BK channels (van Welie and du Lac, 2011). This similar long-term activation of sensitivity and excitation inhibition may be an important cause of neural sensitization following a vestibular lesion.

In VM and PPPD, neural sensitization may manifest as amplified perception of normal sensory input. When sensory gating deficits and abnormal cortical excitability are superimposed, minimal sensory input (such as normal-intensity light, everyday environmental noise, and physiological postural sway) can be perceived as intense stimuli, triggering or exacerbating vestibular symptoms. Emotional factors enhance the sensitivity of sensory processing loops via the descending facilitatory pathway, further exacerbating this process. VM patients often present with spontaneous vertigo, head movement-induced vertigo, and intolerance to visual stimuli (Huang et al., 2020). Patients with PPPD exhibit persistent dizziness, symptom exacerbation when standing, and sensitivity to complex visual environments (Staab, 2020).

### 4.2 Multisensory integration abnormality

After feeling dizzy, abnormal multisensory integration makes the nerves more sensitive. When the head rotates, the vestibular semicircular canals detect angular acceleration, cervical proprioception detects the head-trunk relative position, and the visual system detects retinal image slip; together, these three constitute a complete perception of head movement. When different sensory channels provide conflicting information, the central nervous system must resolve the sensory conflict. Including inhibiting input from unreliable channels, increasing the weight of reliable channels, or reinterpreting sensory information to achieve consistency. When these mechanisms fail, sensory conflict leads to vertigo, nausea, and postural instability. Following a vestibular lesion, the proportion of A-type and B-type neurons in the ipsilateral and contralateral vestibular nuclear complex shifts: the contralateral side exhibits more “B-like” characteristics (increased excitability), and the ipsilateral side exhibits more “A-like” characteristics (enhanced inhibition) (Lacour et al., 2016). Furthermore, after UL, the postsynaptic inhibition of glutamatergic synaptic strength induced by UL, as reflected by a decreased AMPA/NMDA ratio in the ipsilateral MVN, is reversed within 24 h, which may be due to the upregulation of Ca2+-permeable AMPA receptors. The probability of presynaptic glutamate release, as indicated by the reduced frequency of spontaneous excitatory postsynaptic currents, was not fully compensated during this period (Zhang et al., 2025). Furthermore, the cerebellar flocculus and nodulus, by receiving inputs from the vestibular and visual systems (such as retinal slip error signals), drive synaptic plasticity in the VOR pathway to finely adjust reflex gain and timing (Cullen, 2023). For example, the cerebellum regulates the inhibitory effect of Purkinje cells on the vestibular nucleus through mechanisms such as long-term depression (LTD), thereby influencing the VOR gain. A study on UL in mice found that the activity patterns of unipolar brush cells in the ipsilateral cerebellar flocculus were altered, indicating their involvement in vestibular compensation (Liu et al., 2023). The density and morphology of dendritic spines in the ipsilateral MVN return to baseline levels within 6 to 24 h after UL.

Multisensory integration abnormalities are also supported by imaging evidence (Table 2). In patients with unilateral vestibular neuritis, resting-state functional connectivity analysis revealed decreased functional connectivity in the contralateral parietal lobe (intraparietal sulcus, supramarginal gyrus), and as vestibular-induced disability decreased, the functional connectivity gradually increased (Lacour et al., 2016). This indicates that the re-establishment of sensory reweighting involves the reorganization of extensive cortical networks. VBM analysis reveals that gray matter volume in the inferior parietal lobule (IPL) and hippocampus is significantly reduced in patients with PPPD, and is negatively correlated with disease course, suggesting that as the disease progresses, the key brain regions required for multisensory integration undergo structural depletion (Wurthmann et al., 2017). Further analysis using surface-based morphometry (SBM) found that patients with PPPD exhibited significantly reduced local gyrification index (LGI) in the bilateral posterior insular cortex, supramarginal gyrus, and posterior superior temporal gyrus–regions that serve as critical hubs for the convergence of vestibular, somatosensory, and visual information. rs-fMRI analysis revealed that PPPD patients had significantly reduced functional connectivity between the left hippocampus and the bilateral central operculum, posterior operculum, and right insula (Lee et al., 2018), indicating that information transmission between the Vestibular cortex and the spatial memory system is impaired. Another rs-fMRI study found that, compared with the healthy control group, functional connectivity between the left thalamus and the left anterior cingulate cortex, bilateral insula, and right supplementary motor area was reduced in patients with VM. whereas functional connectivity between the right thalamus and the left insula and anterior cingulate cortex was increased (Chen et al., 2022). Significantly decreased regional homogeneity (ReHo) in the left middle temporal gyrus (MTG) and left middle occipital gyrus (MOG) (Liu et al., 2025). In rs-fMRI during simulated vertical self-motion, the activation of the anterior wall of the central insular sulcus (the core region of the parietoinsular vestibular cortex, PIVC) in patients with PPPD was significantly weaker than that in the healthy control group, while at the same time, the activation intensity of the primary visual cortex (V1, V2, V3) was significantly positively correlated with the Dizziness Handicap Inventory (DHI) score (Riccelli et al., 2017b). In elderly patients with chronic dizziness, the left inferior fronto-occipital fasciculus (connecting the occipital visual area to the parietal vestibular area) and the left arcuate fasciculus (connecting the superior temporal gyrus to the frontal lobe) show significantly decreased fractional anisotropy (FA) and increased mean diffusivity (MD), the microstructural integrity of white matter fiber tracts is disrupted (Ban et al., 2026). VBM analysis showed that, compared with the control group, patients with VM had reduced gray matter (GM) volume in the left cerebellum, increased GM volume in the left temporal lobe, and selective GM volume increases in frontal and occipital regions (Messina et al., 2017), and the microstructural integrity of gray matter fiber tracts was disrupted . The decrease in regional homogeneity (ReHo) in the middle occipital gyrus of patients with VM showed a marginally significant negative correlation with attack frequency, indicating that visual cortex function declines as disease burden increases (Liu et al., 2025).

**TABLE 2:** Neuroimaging Findings in PPPD and VM.

Disorder	Brain region	Pathological findings	Technique	Significance	DOI
PPPD	Inferior parietal lobule (IPL), Hippocampus	Significantly reduced gray matter volume, negatively correlated with disease duration	VBM (voxel-based morphometry)	Suggests progressive structural depletion of key regions required for multisensory integration	10.1016/j.jpsychores.2017.10.003
PPPD	Bilateral posterior insular cortex, Supramarginal gyrus, Posterior superior temporal gyrus	Significantly reduced local gyrification index (LGI)	SBM (surface-based morphometry)	These regions are critical hubs for vestibular-somatosensory-visual information convergence	10.1007/s11682-018-9900-6
PPPD	Left hippocampus ↔ Bilateral central operculum, Posterior operculum, Right insula	Significantly reduced functional connectivity	rs-fMRI (resting-state fMRI)	Indicates impaired information transmission between vestibular cortex and spatial memory systems	10.1002/hbm.24080
PPPD	Anterior wall of central insular sulcus (PIVC core region)	Markedly decreased activation	rs-fMRI (during simulated vertical self-motion task)	Reduced vestibular multisensory integration	10.3389/fneur.2017.00529
PPPD	Primary visual cortex (V1, V2, V3)	Activation intensity positively correlated with Dizziness Handicap Inventory scores	rs-fMRI (during simulated vertical self-motion task)	Compensatory overactivation of visual cortex	10.3389/fneur.2017.00529
PPPD	Left inferior fronto-occipital fasciculus (occipital visual area ↔ parietal vestibular area), Left arcuate fasciculus (superior temporal gyrus ↔ frontal lobe)	Significantly decreased FA, increased MD	DTI (diffusion tensor imaging)	Disrupted white matter microstructural integrity, impairing visual information transmission	10.2174/1567202623666230914095036
PPPD	Vestibular cortex ↔ Visual cortex, Somatosensory cortex ↔ Visual cortex	Decreased vestibular-visual connectivity; Enhanced somatosensory-visual connectivity	rs-fMRI (pre- and post-visual stimulation)	The normal vestibular-visual coupling pattern is replaced by abnormal somatosensory-visual coupling	10.3389/fneur.2023.1215004
PPPD	Multimodal vestibular cortex (PIVC, posterior insula, etc.)	Decreased local activity and functional connectivity	rs-fMRI	Functional impairment in core regions of vestibular multisensory integration	10.1007/s13139-020-00675-2; 10.3390/jcm10184274
PPPD	Multimodal vestibular cortical regions	Structural abnormalities: reduced cortical folding, decreased gray matter volume	VBM, SBM	Cortical morphological alterations	10.1007/s13139-020-00675-2; 10.3390/jcm10184274
PPPD	Prefrontal cortex (PFC) ↔ Primary visual cortex, Motor areas	Increased functional connectivity	rs-fMRI	Prefrontal overdrive of visual and motor networks	10.3390/jcm10184274
VM	Left thalamus ↔ Left anterior cingulate cortex, Bilateral insula, Right supplementary motor area	Decreased functional connectivity strength	rs-fMRI (seed-based functional connectivity)	Suggests impaired thalamo-vestibular pathways, affecting transmission of vestibular information to sensorimotor cortical areas	10.1007/s00234-021-02777-w
VM	Right thalamus ↔ Left insula, Anterior cingulate cortex	Increased functional connectivity strength	rs-fMRI (seed-based functional connectivity)	May reflect compensatory enhancement of thalamo-visual pathways	10.1007/s00234-021-02777-w
VM	Left middle temporal gyrus (MTG)	Significantly decreased ReHo, negatively correlated with attack frequency (*r* = −0.59, *p* = 0.037)	rs-fMRI (ReHo, regional homogeneity)	MTG is a multisensory cortical hub; its functional decline reflects impaired neural efficiency of vestibular multisensory information processing in VM	10.1002/brb3.70569
VM	Left middle occipital gyrus (MOG)	Significantly decreased ReHo, marginally negatively correlated with attack frequency (*r* = −0.56, *p* = 0.052)	rs-fMRI (ReHo, regional homogeneity)	Visual cortex function attenuates with increasing disease burden, consistent with the theory of impaired vestibular-visual reciprocal inhibition	10.1002/brb3.70569
VM	Left posterior cerebellum	Significantly decreased ReHo; Decreased FC with cingulate cortex	rs-fMRI (ReHo, FC)	The cerebellum is a center for pain modulation and sensorimotor integration; its functional abnormality may participate in the pathogenesis of VM	10.1002/brb3.70569
VM	Left cerebellum	Decreased GM volume	VBM (voxel-based morphometry)	May reflect neural plasticity depletion in vestibular-cerebellar pathways	10.1007/s00415-016-8349-z
VM	Left temporal lobe	Increased GM volume	VBM	May reflect compensatory synaptic reorganization driven by recurrent vestibular-visual conflicts	10.1007/s00415-016-8349-z
VM	Frontal and occipital regions	Selective increases in GM volume	VBM	VM patients exhibit abnormal remodeling of the multisensory integration network	10.1007/s00415-016-8349-z

PPPD, persistent postural-perceptual dizziness; VM, vestibular migraine; VBM, voxel-based morphometry; SBM, surface-based morphometry; rs-fMRI, resting-state functional magnetic resonance imaging; DTI, diffusion tensor imaging; ReHo, regional homogeneity; FC, functional connectivity; GM, gray matter; FA, fractional anisotropy; MD, mean diffusivity; PIVC, parieto-insular vestibular cortex; IPL, inferior parietal lobule; MTG, middle temporal gyrus; MOG, middle occipital gyrus; LGI, local gyrification index.

### 4.3 Clinical manifestations

Neural sensitization maintains a high sensitivity in patients with chronic dizziness even during interictal periods, and the heightened neural afferent activity leads to a competition for trust among multisensory inputs in the central system. Most directly, when proprioceptive and visual sensory inputs are inconsistent, it can trigger or exacerbate dizziness in patients with VM or PPPD (Moaty and Nada, 2023; Rova et al., 2024; Yagi et al., 2024), such as riding in cars, taking elevators. Research found that the incidence of nausea or dizziness in VM patients when reading while riding in a car is as high as 91.2%, higher than that in PPPD patients (Rova et al., 2024). Precisely because the balance perception threshold may be reduced, patients can perceive subtle body movements earlier, yet this paradoxically aggravates their discomfort (Helmchen et al., 2024; Toupet et al., 2023). Neural sensitization reduces the brain’s processing threshold for vestibular, visual, and somatosensory information, thereby readily triggering migraine attacks and their associated vertigo symptoms (Kaski, 2020; Moreno-Ajona, 2026). Overly sensitive receptors transmit high-gain signals, along with mismatched sensory integration, manifesting as increased visual dependence and proprioceptive processing disorder (Lubetzky et al., 2021). The phenomenon of proprioceptive and visual misalignment in patients with VM and PPPD under specific contexts manifests as an abnormality in the integration of visual, vestibular, and proprioceptive inputs. In VM patients, VOR gain is normal, but dynamic visual acuity (DVA) is significantly reduced, indicating a deficit in the integration of proprioception and vision (Kirazli et al., 2025). Vertigo symptoms of VM may sometimes be described as a form of ‘visual dependence’ or visual vertigo (VV), manifesting as an excessive sensitivity to visual stimulus (Toupet et al., 2023). Anxiety levels in patients with PPPD are significantly higher than those in patients with VM, and anxiety levels correlate with reduced Dynamic Visual Acuity (DVA) (Kirazli et al., 2025). Research shows that patients with PPPD in complex visual environments exhibit significantly lower head movement acceleration than healthy control groups, indicating deficits in visual-proprioceptive integration, along with increased visual dependence and proprioceptive processing disorder (Lubetzky et al., 2021). PPPD patients exhibit a higher visual motion perception threshold in visually complex environments, which may lead to anxiety and uncertainty when facing complex visual stimuli. and exhibit an abnormal sensory-perceptual scaling mechanism when perceiving self-motion (Storm et al., 2024), VM patients exhibit abnormal sensitivity in self-motion perception, which may be related to neural sensitization of central vestibular pathways (King et al., 2019). This sensitization may lead VM patients to exhibit lower Motion Perception Thresholds and higher susceptibility to motion sickness when exposed to vestibular stimulation (Storm et al., 2024; Wurthmann et al., 2021), exhibiting higher neural activity under vestibular stimulation (Storm et al., 2025).

## 5 Psychological sensitization

### 5.1 Fear-avoidance model

Psychological stress can induce plastic changes in the vestibular system, such as adjustments in the excitability of vestibular nucleus neurons and synaptic remodeling (Balaban, 2016). This may be a key reason why some patients who experience an acute vestibular event transition to chronic vestibular disorders. Psychological factors can precipitate functional decline, a phenomenon that can be explained by the ‘fear-avoidance model’. The fear-avoidance model was originally developed to explain the mechanism underlying the maintenance of chronic pain. Its central premise is that catastrophic thinking leads to fear of triggering situations, which in turn gives rise to avoidance behavior; this avoidance behavior then results in functional decline and disability, thus creating a vicious cycle. In vestibular disorders, the fear-avoidance model is manifested as follows: after experiencing a vertigo attack, patients develop a fear of provoking movements, e.g., rapid head turns, turning around, believing that these movements will inevitably cause a fall. This fear leads patients to avoid these movements, and long-term avoidance leads to altered proprioceptive input patterns from the neck, trunk, and lower limbs; the altered proprioceptive input, in turn, makes postural control more difficult, thereby confirming the patient’s concern about instability. The key to vestibular rehabilitation exercises appears to be repeated movements and confidence rebuilding (Zhu et al., 2025) to confront this physical and psychological fear-avoidance.

### 5.2 Emotion-vestibular system interaction

Brain regions involved in emotional control are affected following vestibular dysfunction. Studies of patients with chronic unilateral vestibular disorder have found enhanced activity in brain regions associated with the sensorimotor network (including the insula and prefrontal cortex). The insula is a key hub for interoceptive processing, and the prefrontal cortex participates in emotion regulation and cognitive control (Si et al., 2022). The amygdala, which participates in vestibular compensation, (Zwergal et al., 2016) is an important component of the limbic system and plays a core role in emotional processing, particularly in fear and aggressive emotions (Hayes and Northoff, 2011; Robbins and Everitt, 1999). It participates in the initial detection of emotional stimuli and the evaluation of emotional significance (Wang et al., 2024). Vestibular dysfunction activates the stress axis, manifested as upregulation of arginine vasopressin-immunoreactive cells and corticotropin-releasing factor-immunoreactive cells in the paraventricular nucleus, and an increase in dopamine-β-hydroxylase in the locus coeruleus of unilateral vestibular neurectomy (UVN) cats (Lacour et al., 2016). After detecting threat signals, the amygdala sends excitatory projections to the locus coeruleus via the central amygdala. The locus coeruleus releases norepinephrine (NE) throughout the brain, including the vestibular nuclei. NE acts via βα-adrenergic receptors to enhance the spontaneous firing rate of MVN neurons and their sensitivity to peripheral vestibular input (Zhang et al., 2021). In chronic anxiety states, persistent overactivation of the amygdala keeps the locus coeruleus-norepinephrine system at a high output level, thereby chronically elevating the baseline excitability of vestibular neurons and reducing the sensory threshold. Furthermore, Maywald et al. (2023) found that functional connectivity between the prefrontal cortex, the parieto-insular vestibular cortex (PIVC), and the visual cortex was abnormal in patients with PPPD, possibly reflecting insufficient cognitive control over the sensory network. Gorka et al.’s (2019) study supports this hypothesis, indicating that selective serotonin reuptake inhibitor treatment may improve anxiety and emotional symptoms by enhancing top-down regulation of the prefrontal cortex over the amygdala and insula. The discontinuation of serotonin reuptake inhibitors, in turn, increases the burden on the vestibular complex and disrupts neuronal function (Smith and Darlington, 2010). Furthermore, patients with post-traumatic stress disorder (PTSD) often present with vestibular dysfunction, manifesting as vertigo and balance disorder, and the mechanism may be related to the interaction between the vestibular system and the limbic system (O’Neil et al., 2017). Hashimoto et al. (2022) found that, Central Sensitization Inventory (CSI) scores were significantly correlated with Dizziness Handicap Inventory (DHI) scores, Hospital Anxiety and Depression Scale (HADS) scores in 61 patients with PPPD, and regression analysis indicated that, CS severity was an aggravating factor that exacerbated dizziness symptoms in PPPD.

Studies have shown that psychological factors modulate proprioceptive processing, leading to sensory sensitization and abnormal postural control (Riccelli et al., 2017a). In patients with anxiety, a lowered proprioceptive threshold heightens sensitivity to body posture perception, predisposing them to balance disorders (D’Alessandro et al., 2016). Attentional orienting to proprioceptive stimulation can alter processing in the primary sensorimotor cortex, manifested as a decrease in β wave power and an increase in motor evoked potential amplitude (Piitulainen et al., 2021). Patients with PPPD exhibit abnormalities in functional connectivity of brain regions related to visuospatial processing, postural control, and self-perception, while various factors including personality traits (i.e., neuroticism), psychiatric comorbidities (i.e., anxiety and depression), and trigger factors (i.e., peripheral vestibular disorders) appear to modulate brain functional activity and connectivity patterns (Im et al., 2021). rs-fMRI shows that, compared to healthy controls, patients with Persistent PPPD exhibit enhanced activation of the amygdala, anterior cingulate cortex (ACC), and prefrontal lobe during processing of emotionally negative stimuli, as well as enhanced functional connectivity between the amygdala and ACC (Lee et al., 2018; Maywald et al., 2023). VBM also confirmed that anterior cingulate cortex (ACC) gray matter volume in patients with PPPD is negatively correlated with anxiety scores (Wurthmann et al., 2017). Individuals with higher neuroticism scores exhibited significantly increased activation of the left parietal insular vestibular cortex (PIVC) during a simulated self-motion task, along with enhanced functional connectivity between the PIVC and the right amygdala, suggesting that anxiety-related personality traits can influence vestibular-visual integration (Riccelli et al., 2017a). A study of 301 patients with various vestibular disorders showed (Molnár et al., 2022), patients with VM and PPPD had significantly higher total Dizziness Handicap Inventory (DHI) scores and Beck Depression Inventory (BDI) scores than other patients. Patients with VM and PPPD often present with anxiety and depressive symptoms, and psychological factors can exacerbate their vestibular symptoms (Lahmann et al., 2015). Research shows that the vestibular symptoms of patients with VM can gradually evolve into PPPD, especially when accompanied by anxiety and depression (Ma et al., 2022). Approximately 35.6% of patients with chronic vestibular syndrome are diagnosed with PPPD, and some of these patients have a history of VM (Xing et al., 2024). Patients with PPPD exhibit abnormal postural strategies, such as reduced head movement, in complex visual environments, which may be associated with increased visual dependence. Research has found that when patients with PPPD perform dual visual and cognitive tasks, the acceleration of their head movements is significantly lower than that of the healthy control group, indicating a deficit in their multisensory integration mechanism (Lubetzky et al., 2021). A study of 97 patients with vertigo found (Molnár et al., 2022) that the more severe the vertigo handicap, the higher the likelihood of an anxiety state, and the poorer their visuospatial cognition.

## 6 Neural sensitization and psychological sensitization interact

Neural sensitization and psychological sensitization do not operate independently; rather, they form a mutually reinforcing closed loop. On the one hand, neural sensitization lowers the brain’s processing threshold for vestibular, visual, and somatosensory information (Kaski, 2020; Moreno-Ajona, 2026). The lowered threshold increases the frequency and salience of bodily signals in daily life; for example, the subtle sway of normal posture and natural variations in visual scenes may be perceived as abnormal signals. Excessive bodily signals trigger hypervigilance and catastrophic appraisal. On the other hand, the persistent anxiety and attentional bias generated by psychological sensitization enhance the sensitivity of the sensory processing loop through the descending facilitatory pathway (Figure 1). Patients who over-rely on visual input for spatial orientation are more likely to experience long-term vestibular symptoms (Cousins et al., 2014). Patients with anxiety exhibit reduced proprioceptive thresholds and heightened sensitivity to body posture perception (D’Alessandro et al., 2016); Attentional orienting to proprioceptive stimulation can alter processing in the primary sensorimotor cortex, manifested as a decrease in β wave power and an increase in motor evoked potential amplitude (Piitulainen et al., 2021). Patients with PPPD exhibit abnormalities in functional connectivity of brain regions involved in visuospatial processing, postural control, and self-perception, while psychological factors such as anxiety, dependent personality traits, autonomic arousal, and visual dependence (Trinidade et al., 2023), and trigger events appear to jointly modulate these brain functional activities and connectivity patterns (Im et al., 2021). Their interaction manifests clinically as: the initial acute vestibular event triggers neural sensitization, increasing the patient’s sensitivity to visual and proprioceptive stimuli. Persistent somatic discomfort and overreaction to environmental stimuli further trigger or exacerbate anxiety and fear, forming psychological sensitization. Psychological sensitization, in turn, affects central sensory processing through the limbic system and autonomic nervous system, maintaining or exacerbating neural sensitization, causing dizziness symptoms to persist and be difficult to alleviate (Table 3).

**FIGURE 1:**
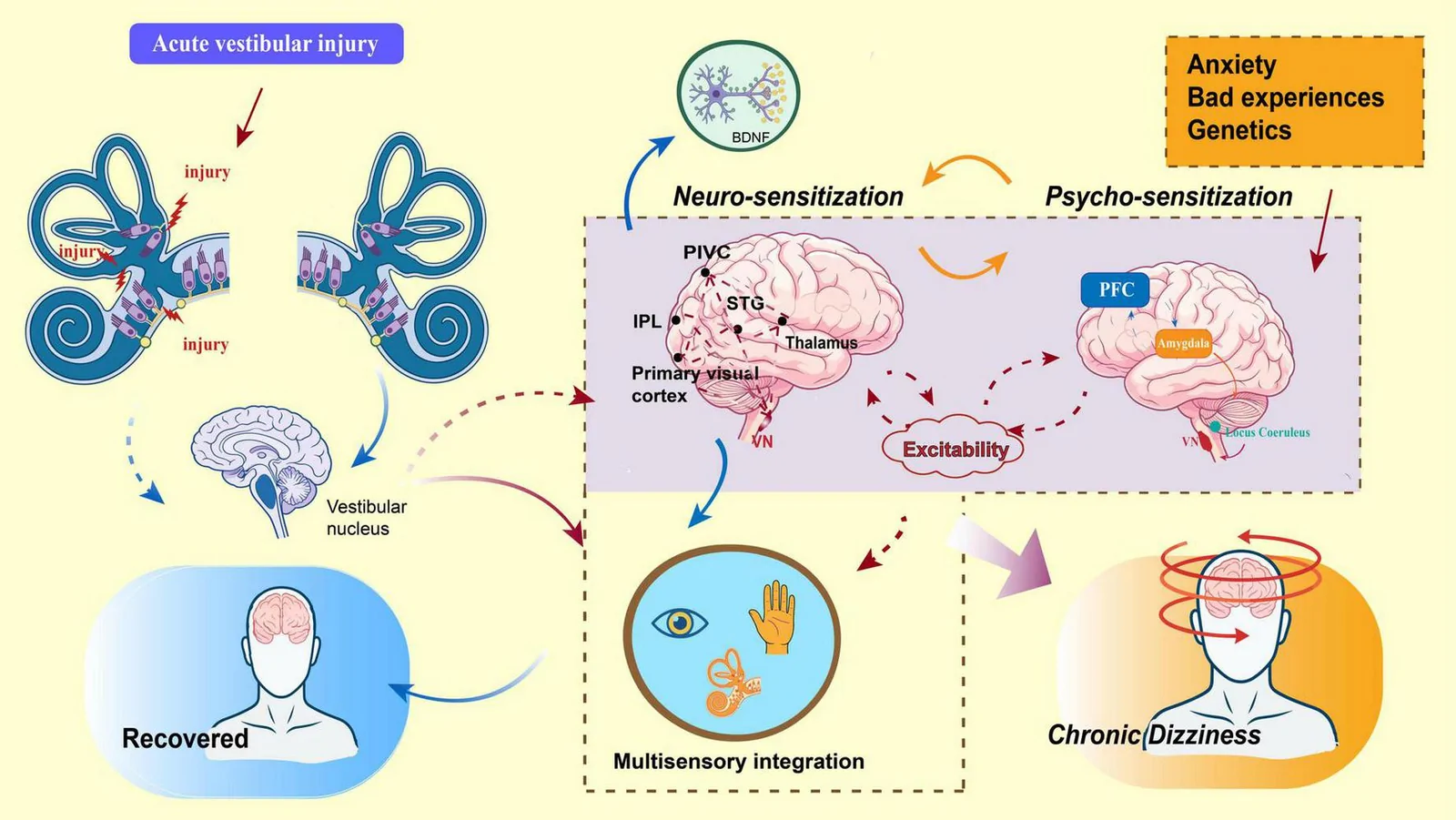
Chronic dizziness develops from acute vestibular injury.

**TABLE 3:** Pathological pathways from acute vestibular events to chronic dizziness.

Pathway	Key mechanistic nodes	Primary drivers	Brain regions/circuits involved	Clinical features	Outcome
Pathway 1: successful compensation	① Rebalancing of resting discharge in bilateral vestibular nuclei ② Adaptive sensory reweighting (moderate increase in visual/proprioceptive weighting) ③ Synaptic plasticity repair	Intrinsic CNS plasticity; No significant psychiatric comorbidity; Good social support	Vestibular nuclei complex, Cerebellar flocculus, Temporo-parietal cortex (short-term reversible remodeling)	Acute symptoms significantly diminish within weeks to months; only minimal discomfort in extreme environments (darkness, uneven ground)	Self-limiting recovery
Pathway 2: neural-dominant sensitization	① Synaptic/neuronal hyperexcitability ② Lowered sensory processing thresholds (visual and proprioceptive hypersensitivity) ③ Abnormal multisensory integration (reduced visual-vestibular coupling; enhanced somatosensory-visual coupling)	Severe initial injury; Persistent sensory conflict (e.g., repeated vehicle travel); Continuous abnormalities in the central nervous system	MVN, PIVC, Insula, Visual cortex (V1–V3), Cerebellum	Overreaction to normal light, daily environmental noise, and physiological postural sway; Reduced dynamic visual acuity; Significant visual dependence	Persistent dizziness
Pathway 3: psycho-dominant sensitization	① Fear-avoidance cycle (catastrophic cognition → avoidance behavior → proprioceptive abnormalities) ② Overactivation of amygdala-locus coeruleus-vestibular nucleus axis ③ Reduced prefrontal top-down regulation of sensory networks	High neuroticism; Pre-existing anxiety/depression; Adverse childhood experiences; Acute-phase hypervigilance	Amygdala, Anterior cingulate cortex (ACC), Prefrontal cortex (PFC), PIVC	Excessive worry about postural instability; Symptoms triggered or aggravated by supermarkets, crowds, and vehicular motion; Significantly elevated DHI scores	Persistent dizziness
Pathway 4: dual-sensitization vicious cycle (final common pathway)	① Neuro-sensitization → increased somatic signals → triggers hypervigilance and catastrophic appraisal ② Psycho-sensitization → enhanced sensory processing gain via descending facilitatory pathways ③ further lowers sensory thresholds → Abnormal hippocampal-amygdala-PIVC functional connectivity	Overlap of factors from Pathways 2 and 3; Genetic susceptibility	Widespread network: PIVC + IPL + Hippocampus + Amygdala + PFC + Visual cortex; Disrupted white matter tract integrity (Inferior fronto-occipital fasciculus, Arcuate fasciculus)	Refractory dizziness/vertigo; Comorbidity or mutual transformation between VM and PPPD; Poor response to anxiolytics, anticonvulsants, and rehabilitation therapy	Refractory chronic dizziness / Chronicity

## 7 Individual differences

For individuals, psychological sensitization leading to the transition from an acute vestibular event to chronic dizziness may be influenced by multiple factors. Neuroticism is the most important personality dimension predicting the functional burden of chronic dizziness. Naudé and Kanji (2026) in a clinical observational study of 247 patients with chronic dizziness, the Eysenck Personality Questionnaire and the Dizziness Handicap Inventory (DHI) were used to assess the relationship between personality traits and functional burden. The results indicated that neuroticism was significantly positively correlated with DHI scores and was the only independent variable that significantly predicted DHI scores, irrespective of diagnostic category and symptom duration. Neuroticism is associated with increased task-dependent functional connectivity between the amygdala and the hippocampus and dorsolateral prefrontal cortex, and with coupling between the amygdala and regions involved in emotion regulation and salience processing, such as the anterior insula and dorsal cingulate cortex. This heightened connectivity may account for the persistence of negative emotions and emotion regulation difficulties observed in neurotic individuals (Meiering et al., 2026). Neuroticism is positively correlated with altered functional coupling between the basolateral subregion of the amygdala and the anterior insular cortex, dorsal anterior cingulate cortex, and anterior cingulate cortex, suggesting that individuals with high neuroticism may be more prone to hypervigilance after acute stress (Wang et al., 2018). Adverse childhood experiences can lead to long-term alterations in HPA axis function, thereby affecting an individual’s stress response (Blecker et al., 2025; Gong et al., 2025). Adverse events experienced by the mother during childhood can influence behavioral problems in her offspring through functional alterations of the HPA axis. This transgenerational effect may be mediated by the mother’s HPA axis function during pregnancy and is modulated by social support (Thomas et al., 2018; Thomas-Argyriou et al., 2021). Genetic factors also contribute to individual differences. The BDNF Val66Met polymorphism (rs6265) is one of the most extensively studied functional genetic variants. Met allele carriers exhibit reduced activity-dependent secretion of BDNF, which affects hippocampal synaptic plasticity and fear extinction learning (Lugo-Candelas et al., 2018). Catechol-O-methyltransferase (COMT) is an enzyme involved in dopamine metabolism. The COMT Val158Met polymorphism affects dopamine levels in the prefrontal cortex, and when facing the same stress, individuals with different genotypes exhibit different adaptive responses (Buckert et al., 2012). It is the 5-hydroxytryptamine transporter gene-linked polymorphic region (5-HTTLPR) polymorphism that affects the expression level of the 5-HT transporter; carriers of the short allele (S) have reduced 5-HT reuptake efficiency, which is associated with increased anxiety susceptibility (Loewenstern et al., 2019). The 5-HTTLPR polymorphism also influences the stress response following stress (Kuhn et al., 2021). Psychological factors and hypersensitivity to self-motion may appear before or after the disease, and for individuals with certain personality traits, specific genetic profiles, and sensitive proprioception, unique talents may exist in some domains. But dizziness (including vertigo) affects approximately 15% to over 20% of adults each year (Neuhauser, 2016), and for sensitive individuals who are prone to hypervigilance and constant worry, the onset of chronic dizziness seems predictable.

## 8 Individualized treatment

Neural sensitization and psychological sensitization are intertwined. In the treatment of chronic dizziness, both aspects are addressed concurrently. Vestibular rehabilitation, sensory reweighting training, and confidence building are considered effective interventions; these methods alleviate symptoms by improving patients’ sensory integration ability and psychological state. Medications are primarily selective serotonin reuptake inhibitors (SSRIs) and serotonin-norepinephrine reuptake inhibitors (SNRIs). Trinidade and Goebel (2018), in addition to basic modulation of serotonin, they also activate descending inhibitory pathways, which is crucial for regulating sensory input, thereby reducing neural sensitivity. Browne (2025), especially in regions such as the raphe nuclei (Kumar and Itagi, 2021), further emphasizing their role in reducing neural sensitivity and psychological aspects. Vestibular rehabilitation exercises (VRT), through repeated exposure to motion and visual stimuli that provoke dizziness, gradually reduce the sensitivity of neural input, while breaking avoidance behaviors, thereby reducing levels of psychological sensitization and neural sensitization.

From a more individualized perspective, if a patient exhibits more sensory processing abnormalities and autonomic dysfunction—tendencies related to psychological sensitization—consider adding transcranial electrical stimulation; transcranial alternating current stimulation of the dorsal prefrontal cortex (PIVC) can effectively reduce vestibular responses and cortical excitability (McCarthy et al., 2025, 2026). Transcranial direct current stimulation (tDCS) targeting the parieto-insular vestibular cortex can also alleviate vestibular-related symptoms, such as motion sickness, by reducing cortical excitability in this region (Li et al., 2025). This Excitation inhibition helps balance sensory reweighting, thereby optimizing Multisensory integration. Furthermore, antiepileptic drugs such as pregabalin and topiramate, among others, have been shown to inhibit calcium influx, thereby reducing neural excitability (Belete, 2023). These mechanisms are particularly important when considering their application in diseases characterized by abnormal neural discharge, including dizziness associated with neural hyperexcitability (Ng et al., 2025). Although evidence for its specific application in dizziness is still accumulating, its mechanisms in chronic pain and central sensitization (CS) syndrome offer valuable insights. Patients with a strong tendency toward psychological sensitization often exhibit high scores on the Dizziness Handicap Inventory (DHI), Anxiety and Depression Scale scores, and pronounced fear-avoidance behavior. The addition of cognitive behavioral therapy (CBT) may be beneficial.

## 9 Discussion

Compensation after a vestibular lesion increases visual dependence. In a fear-avoidance state, visual dependence persists, leading to an overreaction to minor external fluctuations, which transforms the acute vestibular event into a chronic condition. The degree of psychological abnormality may determine the direction of this transition. Patients with PPPD and VM who have the most pronounced emotional disturbances are more sensitive (Young et al., 2021). Previous research has found that patients with pre-existing psychiatric disorders or dependent personality traits are more likely to develop chronic dizziness. Patients with higher levels of resilience, sense of coherence, and life satisfaction are less likely to develop secondary functional dizziness 1 year after the onset of a vestibular disorder. Pre-existing neuroticism and related personality traits before the illness, along with high levels of body vigilance (i.e., conscious attention to dizziness) and catastrophic concerns about the causes and consequences of vestibular symptoms in response to an acute vestibular event after the illness, drive the development of chronic dizziness (Trinidade et al., 2023), thereby transforming a transient dizziness that is potentially curable into a chronic condition (Figure 2).

**FIGURE 2:**
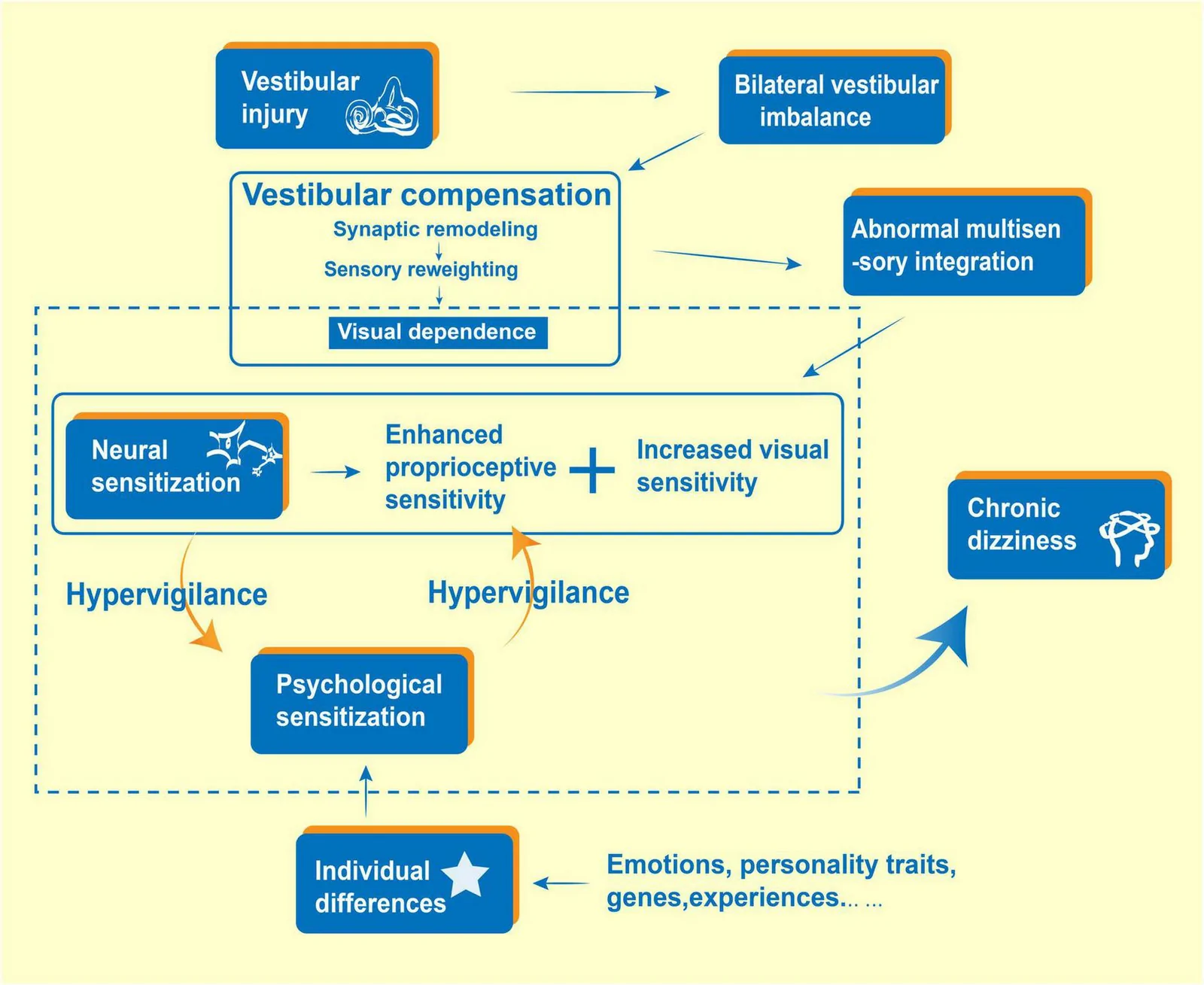
Mechanism diagram.

Similarly, hypersensitivity to precipitating factors (upright posture/walking, self-motion, visual stimuli) demonstrates high sensitivity in identifying patients who subsequently develop PPPD (Kabaya et al., 2022), and the presence of these three precipitating factors in the early stage of the disease likely renders this population more susceptible. For example, patients with PPPD often adopt a rigid postural control strategy and remain hypervigilant to their surroundings, which may result from the combined action of neural sensitization and psychological sensitization (Hashimoto et al., 2022; Waterston et al., 2021). This hypervigilance drives patients to constantly scan the environment for potential threats, even when none exist. For example, they may exhibit abnormal sensitivity and discomfort to dense visual information on supermarket shelves, moving crowds, or slight motion when traveling in vehicles. This behavioral pattern is both a manifestation of neural sensitization and a reflection of underlying anxiety and fear. Unlike PPPD, VM is characterized not only by episodic vertigo but also by migraine symptoms, including cortical spreading depression (CSD) and central nervous system hyperexcitability (Kaski, 2020; Moreno-Ajona, 2026). Can lead to transient inhibition of neuronal function, affecting sensory processing (Ma et al., 2022), such as transient visual distortions or a sense of vertigo. Abnormal excitability of the central nervous system and neurotransmitter imbalances (e.g., 5-hydroxytryptamine and glutamate) may simultaneously affect migraine pathways and emotion regulation networks (Ma et al., 2022).

The dual sensitization model, as a theoretical framework, is inevitably a simplification. Some individuals with severe health anxiety develop chronic dizziness-like symptoms even in the absence of a clear peripheral vestibular lesion, suggesting that in certain subgroups, psychological sensitization alone may be sufficient to drive symptoms. At the molecular level, the boundary between neural sensitization and psychological sensitization becomes blurred. Inflammatory cytokines and stress hormones can directly modulate synaptic plasticity, while the expression of neurotrophic factors such as BDNF is regulated by environmental and psychological states. Furthermore, most of the cited neuroimaging studies are cross-sectional in design with small sample sizes, thus limiting causal inference. Animal models, especially the UL model, can provide mechanistic evidence; however, they are too extreme. The operational definitions of neural sensitization and psychological sensitization proposed in this review have not yet undergone systematic psychometric validation and lack standardized clinical cutoff values.

## 10 Conclusion

In summary, the transition from an acute vestibular event to chronic dizziness is not determined solely by the degree of peripheral injury; rather, it depends on the interplay between central neural sensitization and psychological sensitization. Neural sensitization manifests as excitatory gain at the synaptic and neuronal level, reducing the processing threshold for visual, proprioceptive, and vestibular information. Psychological sensitization, in turn, manifests as a fear-avoidance cycle and hypervigilance. An initial acute vestibular event may trigger neural sensitization, causing an increase in the patient’s sensitivity to visual and proprioceptive stimulation. This persistent somatic discomfort and overreaction to environmental stimuli can further trigger or exacerbate anxiety and fear, leading to psychological sensitization. Psychological sensitization, in turn, influences the central nervous system via the autonomic nervous system and limbic system, maintaining or exacerbating neural sensitization, thus causing dizziness symptoms to persist and become difficult to relieve. The two form a mutually reinforcing vicious cycle: abnormal somatic signals generated by neural sensitization trigger psychological sensitization, and psychological sensitization, through Limbic system modulation, perpetuates and consolidates neural sensitization.
